# Etiology and Pathogenesis of Latent Autoimmune Diabetes in Adults (LADA) Compared to Type 2 Diabetes

**DOI:** 10.3389/fphys.2019.00320

**Published:** 2019-03-26

**Authors:** Sofia Carlsson

**Affiliations:** Institute of Environmental Medicine, Karolinska Institutet, Stockholm, Sweden

**Keywords:** LADA, type 2 diabetes, lifestyle, epidemiology, prevention

## Abstract

As the heterogeneity of diabetes is becoming increasingly clear, opportunities arise for more accurate assessment of factors influencing disease onset, which may lead to more efficient primary prevention. LADA – latent autoimmune diabetes in adults – is a common, hybrid form of diabetes with features of both type 1 and type 2 diabetes. This review aims to summarize current knowledge on the pathophysiological and etiological overlap and differences between LADA and type 2 diabetes, discuss similarities between LADA and type 1 diabetes and point at future research needs. Studies conducted to date show a clear genetic overlap between LADA and type 1 diabetes with a high risk conferred by variants in the human leukocyte antigen (HLA) region. In contrast, data from the limited number of studies on lifestyle factors available indicate that LADA may share several environmental risk factors with type 2 diabetes including overweight, physical inactivity, alcohol consumption (protective) and smoking. These factors are known to influence insulin sensitivity, suggesting that insulin resistance, in addition to insulin deficiency due to autoimmune destruction of the beta cells, may play a key role in the pathogenesis of LADA. Moreover, this implies that onset of LADA, similar to type 2 diabetes, to some extent could be prevented or postponed by lifestyle modification such as weight reduction and increased physical activity. The preventive potential of LADA is an important topic to elucidate in future studies, preferably intervention studies.

## Introduction

Diabetes is the world’s fastest growing disease and a major threat to public health worldwide (NCD Risk Factor Collaboration [NCD-RisC], 2016). Since it is a chronic disease, first and secondary prevention is key in reducing its burden on individuals and society. A prerequisite for efficient primary prevention is knowledge about factors influencing disease onset. Diabetes is a more heterogeneous disease than the crude subdivision into type 1 and type 2 diabetes implies (Tuomi et al., 2014; Leslie et al., 2016; Ahlqvist et al., 2018); with a finer classification of diabetes based on differences in pathophysiology, opportunities arise for more accurate assessment of factors influencing disease onset and prognosis. This may allow us to identify etiological factors potentially obscured in analysis of heterogeneous patient groups with different pathogenesis and help us identify modifiable factors which may eventually lead to improved primary prevention. LADA – latent autoimmune diabetes in adults – is a common but understudied form of diabetes with features of both type 1 and type 2 diabetes. The aim of this review is to summarize findings regarding the role of environmental and genetic factors in the etiology of LADA, discuss potential etiological and pathophysiological overlap between LADA and type 2 diabetes, point at similarities between LADA and type 1 diabetes and the preventive potential of LADA.

## Lada

In 1977, Irvine et al. showed that 11% of individuals initially diagnosed with type 2 diabetes have antibodies against the insulin producing beta-cells, which is a characteristic of type 1 diabetes and indicative of an autoimmune pathogenesis (Irvine et al., 1977). The term LADA was introduced in 1993 by Tuomi et al. to describe this subgroup of patients that seems to share phenotypical features with type 2 diabetes and immunological features with type 1 diabetes (Tuomi et al., 1993). LADA has been estimated to account for 3–12% (Turner et al., 1997; Tuomi et al., 1999; Takeda et al., 2002; Zinman et al., 2004; Buzzetti et al., 2007; Maioli et al., 2010; Hawa et al., 2013; Rasouli et al., 2013b; Zhou et al., 2013; Maddaloni et al., 2015) of all diabetes in adults, being more frequent in Europe (Turner et al., 1997; Tuomi et al., 1999; Buzzetti et al., 2007; Maioli et al., 2010; Hawa et al., 2013; Rasouli et al., 2013b) than in other parts of the world including Asia (Takeda et al., 2002; Zhou et al., 2013; Maddaloni et al., 2015) and North America (Zinman et al., 2004). The majority of LADA studies conducted to date comes from European countries but an increasing number of studies are being conducted also outside Europe (Mishra et al., 2018).

There is no uniform agreement on the definition of LADA but diagnosis is usually based on three criteria (Fourlanos et al., 2005; Naik et al., 2009); (1) adult age at onset, (2) islet autoantibodies as a marker of autoimmune activity, (3) insulin independence. However, the exact application of these criteria varies; Onset should be in adulthood but the actual age limit varies, although 30 or 35 years is most commonly used. The slow onset that distinguishes LADA from type 1 diabetes with adult onset is typically defined as absence of insulin treatment during the first 6 or 12 months following diagnosis, but this criterion has been questioned since it is subjective and depends on the judgment of the treating physician (Brophy et al., 2008). One alternative is to use fasting C-peptide levels as an indicator of remaining insulin secretion and a “latent” onset (Takeda et al., 2002; Rasouli et al., 2016). The least controversial criterion is the assessment of autoantibodies as a marker of the autoimmune activity that separates LADA from type 2 diabetes. Glutamic acid decarboxylase antibodies (GADA) are most frequently used due to the fact that this antibody is far more common in patients with adult onset autoimmune diabetes than other autoantibodies often found in children with type 1 diabetes (Tuomi et al., 1999; Sørgjerd et al., 2012; Hawa et al., 2013).

## Pathogenesis

### Type 1 and Type 2 Diabetes

The main pathophysiological features of type 2 diabetes are insulin resistance in skeletal muscle, liver and adipose tissue, together with impaired insulin secretion (DeFronzo et al., 2015). Insulin resistance reduces peripheral glucose uptake and stimulates hepatic glucose output which leads to elevated blood glucose levels. The ensuing hyperglycemia increases the demand on the beta-cells for a compensatory rise in insulin secretion. This may consequently exhaust the beta-cells and lead to a progressive loss of beta-cell function, resulting in insulin deficiency and subsequent diabetes (DeFronzo et al., 2015). Type 1 diabetes on the other hand seems to result from a distinct pathophysiological process; its main feature is insulin deficiency which is caused by autoreactive T-cells of the immune system that destroy the pancreatic beta-cells. The autoimmune reaction leads to a progressive loss of functional beta-cell mass and declining insulin production (Burrack et al., 2017).

### LADA

Similar to type 1 diabetes, LADA patients display autoantibodies which is indicative of an autoimmune pathogenesis. However, the autoimmune process seems to be milder and the progression of beta-cell failure slower; this is evidenced by the fact that LADA patients consistently display higher levels of C-peptide as indicator of insulin secretion (Carlsson et al., 2000; Hosszúfalusi et al., 2003; Hernandez et al., 2015) and furthermore, they are not insulin-requiring for some time following diagnosis. Compared to patients with type 2 diabetes, those with LADA have less insulin secretion (Turner et al., 1997; Carlsson et al., 2000; Hernandez et al., 2015; Hjort et al., 2018b) and progress to insulin dependence faster (Turner et al., 1997; Zampetti et al., 2014). As an example, data from the United Kingdom Prospective Diabetes Study (UKPDS) showed that among individuals initially diagnosed with type 2 diabetes, 84% of those who were GADA positive at diagnosis progressed to insulin dependence within 6 years compared to 14% of antibody negative individuals (Turner et al., 1997). Similar to what is seen in type 1 diabetes (Insel et al., 2015), autoimmunity seems to be the first pathological symptom in LADA; data from the prospective HUNT Study show that 64% of LADA patients where GADA positive several years prior to diagnosis (Sørgjerd et al., 2012) and similar findings were reported by Lundgren et al. in the Finnish Botnia Study (Lundgren et al., 2010).

Whereas type 1 diabetes typically is characterized by a clustering of different islet autoantibodies (Regnell and Lernmark, 2017), LADA patients tend to be positive primarily for GADA (Tuomi et al., 1999; Sørgjerd et al., 2012; Hawa et al., 2013); In Action LADA, a European multicenter study, 90% of the antibody positive patients were positive for GADA, whereas only 10% could be detected merely through antibodies to insulinoma-associated antigen-2 (IA-2A) or zinc transporter 8 (ZnT8A) (Hawa et al., 2013). Moreover, multiple antibodies were found in 24% of antibody positive patients in Action LADA (Hawa et al., 2013) and in 10% of LADA patients in the Norwegian HUNT Study (Sørgjerd et al., 2012). This indicates that at least for research purposes, it is enough to measure GADA in order to separate LADA from type 2 diabetes. The level of GADA is inversely related to C-peptide levels as a marker of insulin secretion (Radtke et al., 2009) and GADA may therefore serve as an indicator not only of the presence of autoimmune activity but also to some extent, of the magnitude of such activity.

The fact that LADA patients have more insulin production than patients with type 1 diabetes at time of diagnosis indicates that other mechanisms besides autoimmune destruction of the beta-cells may play a role in the pathogenesis. In line with this, it has been shown that LADA patients display insulin resistance (Carlsson et al., 2000; Behme et al., 2003; Chiu et al., 2007; Juhl et al., 2014; Hjort et al., 2018b), although not as pronounced as in type 2 diabetes (Zinman et al., 2004; Lundgren et al., 2010; Hjort et al., 2018b), which has been attributed to differences in BMI (Chiu et al., 2007; Juhl et al., 2014). The relative contribution of insulin resistance to the development of LADA may depend on the degree of underlying autoimmunity; both Swedish and Norwegian data show that LADA patients with less autoimmune activity as indicated by low GADA levels, tend to be more insulin resistant than those with high GADA levels (Hjort et al., 2018b).

## Etiology

### Genetic Factors

In type 1 diabetes, the strongest genetic influence is conferred by genes in the HLA complex which are responsible for approximately half of the genetic susceptibility (Noble et al., 1996). In contrast, the genetic influence on type 2 diabetes risk seems to be spread all across the genome and attributed to a large number of common, genetic variants – each contributing a small amount to heritability of the disease (Fuchsberger et al., 2016). The strongest effect is conferred by variants in the transcription factor 7-like 2 (*TCF7L2*) gene, which is associated with a 30% risk increase. The majority of known type 2 diabetes-related genetic risk variants are associated with insulin secretion rather than insulin resistance (Fuchsberger et al., 2016). Similar to type 1 diabetes, LADA is closely linked to genes in the HLA complex, and furthermore, the highest risk is seen in carriers of the HLA haplotypes HLA-DRB1^∗^04-DQB1^∗^03:02 and HLADRB1^∗^ 03:01-DQB1^∗^02:01, which also confer the highest risk of type 1 diabetes (Desai et al., 2007; Andersen et al., 2010; Pettersen et al., 2010; Mishra et al., 2017). The HLA genes encode the major histocompatibility complex (MHC) that regulates the immune system, consequently these findings thus point to a strong involvement of the immune system in the pathogenesis of LADA (Andersen and Hansen, 2018). In addition, LADA is linked with type 1 diabetes associated variants outside of the HLA region including *PTPN22*, *INS*, and *SH2B3* (Mishra et al., 2017). A genetic overlap with type 2 diabetes has also been reported; the most consistent finding is an association with type 2-associated variants of *TCF7L2* (Cervin et al., 2008; Andersen et al., 2014). This evidence comes primarily from candidate gene studies of which the largest was based on 978 LADA cases of European descent (Mishra et al., 2017) however, recently the first GWAS study on LADA was published including pooled data from 2634 patients (Cousminer et al., 2018). This study confirms that the genetic basis of LADA primarily resembles that of type 1 diabetes, but also includes genetic variants associated with type 2 diabetes. Importantly, studies conducted to date consistently show that the excess risk of LADA conferred by HLA genotypes is far stronger than the risk observed for type 2-associated genetic variants (Andersen and Hansen, 2018). In support hereof, a recent study of family history of diabetes indicated that the risk of LADA is increased six-fold in individuals with family history of type 1 diabetes compared to 2-fold in those with family history of type 2 diabetes (Hjort et al., 2017a).

### Lifestyle

#### Type 1 and Type 2 Diabetes

Strong support for a role of environmental factors in the etiology of autoimmune diabetes is provided by the world wide rise in incidence of type 1 diabetes in children (Diamond Project Group, 2006; Mayer-Davis et al., 2017), which proposedly reflects an increasingly diabetogenic environment rather than changes in the genetic makeup of the population. Still, the environmental triggers of autoimmunity and type 1 diabetes has proved difficult to map. Associations with a number of lifestyle factors have been reported, including exposure to enterovirus, several dietary factors, weight gain and psychological stress (Rewers and Ludvigsson, 2016). Nevertheless, attempts to replicate findings across studies have often failed and intervention studies have been largely unsuccessful in preventing type 1 diabetes in children (Skyler, 2013).

More is known about the etiology of type 2 diabetes; the risk is closely linked to a number of unhealthy lifestyle factors and among those, excess weight is the strongest risk factor; obesity is associated with a seven-fold increased risk according to a meta-analysis based on 18 prospective cohort studies (Abdullah et al., 2010). Low birth weight, proposedly reflecting fetal malnutrition, is also associated with an increased risk of type 2 diabetes (Whincup et al., 2008). In contrast, physical activity is associated with a reduced risk (Smith et al., 2016), whereas sedentary time (Biswas et al., 2015) and hours of TV viewing (Patterson et al., 2018) is positively associated with incidence of type 2 diabetes. Smokers have excess risk that increases in a dose-response manner by number of cigarettes (Pan et al., 2015). Several dietary factors have also been linked to type 2 diabetes even after adjustment for BMI; Food groups associated with a reduced risk include whole grain (Schwingshackl et al., 2017), fruit and vegetables (Schwingshackl et al., 2017), alcohol (moderate vs. no intake) (Li et al., 2016) and coffee (Schwingshackl et al., 2017), while an increased risk is associated with consumption of sugar-sweetened beverages (Schwingshackl et al., 2017), and red and processed meat (Schwingshackl et al., 2017). The preventive potential of type 2 diabetes seems to be substantial; a recent study based on the China Kadoorie Biobank including >400 000 participants estimated that the combination of healthy BMI, waist-hip ratio (WHR) and diet together with non-smoking could prevent 3/4 of all cases of type 2 diabetes (Lv et al., 2017). Similar findings have been reported in Europe (Laaksonen et al., 2010) and in different ethnic groups in the United States (Steinbrecher et al., 2011). The biological mechanism linking the majority of these lifestyle factors to type 2 diabetes primarily involves promotion of insulin resistance or beneficial effects on insulin sensitivity (Petersen and Shulman, 2006). Since LADA, in addition to autoimmunity, seems to be characterized by insulin resistance it may be hypothesized that environmental or lifestyle factors known to promote insulin resistance may also increase the risk of LADA.

#### LADA

There are few studies on the influence of lifestyle factors on the risk of LADA (Carlsson, 2018) and the reason for this is most likely lack of data. Prerequisites for such studies are; (a) information on incident cases of LADA which requires antibody testing in order to separate LADA from type 2 diabetes, (b) a suitable non-diabetic population for comparison, (c) detailed information on lifestyle factors from the time before diagnosis, and (d) enough patients for viable analyses. Characteristics of some of the largest individual LADA studies to date are presented in Table 1. From the table it is evident that few studies fulfill these criteria and consequently, the risk of LADA in relation to lifestyle factors has so far only been investigated with data from the Norwegian HUNT (Nord-TrØndelag Health) Study (Krokstad et al., 2013) which is the world’s only prospective cohort study of LADA (Hjort et al., 2018b), and the Swedish ESTRID (Epidemiological Study of Risk Factors for LADA and Type 2 diabetes) Study (Rasouli et al., 2016) which is a case-control study with incident cases recruited from the ANDIS (All new diabetics in Scania) biobank (Ahlqvist et al., 2018) and incidence density sampled controls (Vandenbroucke and Pearce, 2012). Both studies are population-based and according to the most recent publication based on these data bases, the number of LADA patients is 147 in the HUNT Study and 425 in the ESTRID Study (Hjort et al., 2018b). With data from these two studies, it is possible to calculate separate risk estimates for LADA and type 2 diabetes using the same reference population, exposure and confounding assessment. Unfortunately, patients with type 1 diabetes are not included in the ESTRID-study, and the HUNT-study has too few incident cases of adult onset type 1 diabetes for viable analyses. For that reason, comparisons between LADA and type 1 diabetes has to be based on findings in children from other cohorts. There are additional, larger LADA studies based on pooled data (e.g., 40 and 46) that are not described in Table 1, however, neither of these studies report information on lifestyle factors. ESTRID and HUNT studies have been approved by ethical boards for medical research in Sweden and Norway and all participants gave informed consent.

**Table 1:** Description of some of the largest individual LADA studies to date.

Reference	Study	Setting	Time period	Study design	Study participants	No. cases	LADA definition	Time since diagnosis	Genetic information	Lifestyle information
Andersen et al., 2010	Botnia	Finland	1990	Cross-sectional (with prospective part)	Patients recruited through primary care and their family members	213	≥35 years, GADA positive, no insulin ≤6 months	Not mentioned	Yes	None presented
Buzzetti et al., 2007	NIRAD	Italy	2001–2004	Cross-sectional with prevalent cases	Patients recruited through 83 diabetes clinics	193	Adult onset, GADA or IA-2A positivity, non-insulin requiring,	6 months to 5 years	Yes	None presented
Hawa et al., 2013	Action LADA	Europe	2004–2007	Cross-sectional with prevalent cases	Patients recruited through primary care or hospital settings.	598	30–70 years, GADA/ IA-2A or ZnT8A positivity, no insulin ≤6 months	<5 years	None presented	None presented
Hjort et al., 2018b	ESTRID	Sweden	2010	Case-control with incident cases and matched controls	Patients recruited through primary care and controls from population registry	425	≥35 years, GADA positive, remaining insulin production indicated by C-peptide	Median 5 months	Yes	Yes
Maddaloni et al., 2015	ICLDC database	United Arab Emirates	2013	Cross-sectional with prevalent cases	Patients attending the Imperial College London Diabetes Centre	437	>30 years, GADA or IA-2A positivity, non-insulin requiring	≤10 years	None presented	None presented
Maioli et al., 2010	–	Sardinia	2000–2005	Cross sectional	Patients recruited through five diabetes units	251	35–70 years, GADA positivity, no insulin ≤8 months	0–5 years	Yes	None presented
Rasouli et al., 2013b	HUNT	Norway	1984–2008	Prospective cohort study with incident cases	General population in the County of Nord-TrØndelag in Norway	140	≥35 years, GADA positivity	Incident cases	Yes	Yes
Takeda et al., 2002	Ehime study	Japan	1998–99	Cross-sectional study with prevalent cases	Patients recruited through hospitals	97	>20 years, GADA positivity, remaining insulin secretion indicated by C-peptide	Mean > 10 years	Yes	None presented
Turner et al., 1997	UKPDS	United Kingdom	1977–1991	Cross-sectional	Patients recruited through 23 centers	430	25–65 years, GADA/ autoantibodies toislet-cell cytoplasm (ICA) positivity	“newly diagnosed”	No	None presented
Wod et al., 2018	–	Denmark	1997–2013	Cross-sectional with prevalent cases	Patients referred to the University hospital in Odense	327	≥18 years, GADA positivity, remaining insulin secretion indicated by C-peptide	Not mentioned	None presented	None presented
Zhou et al., 2013	LADA China Study	China	2006–2010	Cross-sectional with newly diagnosed cases	Patients recruited through 46 hospitals	287	≥30 years, GADA positive, non-insulin requiring	<1 year	Yes	None presented
Zinman et al., 2004	ADOPT	United States, Europe	2000–2002	Cross-sectional with prevalent cases	Patients recruited through 488 center	174	Adult onset, GADA positivity, non-insulin requiring	3 years	None presented	None presented


##### Environmental factors in LADA associated with insulin resistance and type 2 diabetes

**FIGURE 1:**
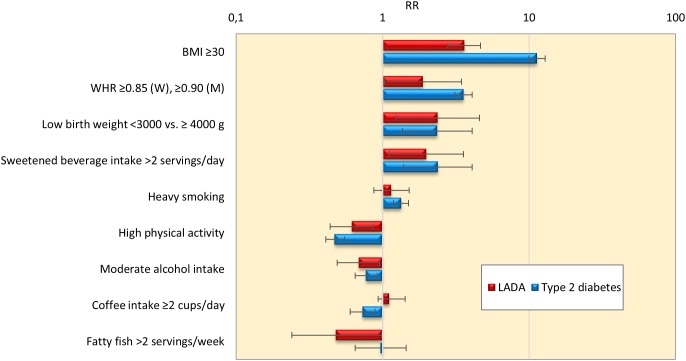
Relative risk and 95% confidence interval for LADA and type 2 diabetes in relation to lifestyle factors. Results from ESTRID and HUNT studies. Estimates for low birth weight, sweetened beverages, coffee intake and fatty fish are based on data from the ESTRID Study (extracted from Löfvenborg et al., 2014, 2016; Hjort et al., 2015; Rasouli et al., 2018); estimates for BMI, smoking, physical activity and alcohol intake are based on pooled data from ESTRID and HUNT studies (extracted from Rasouli et al., 2013a,b, 2014, 2016; Hjort et al., 2018a,b); estimates for WHR is based on the HUNT Study (Hjort et al., 2018b).

**FIGURE 2:**
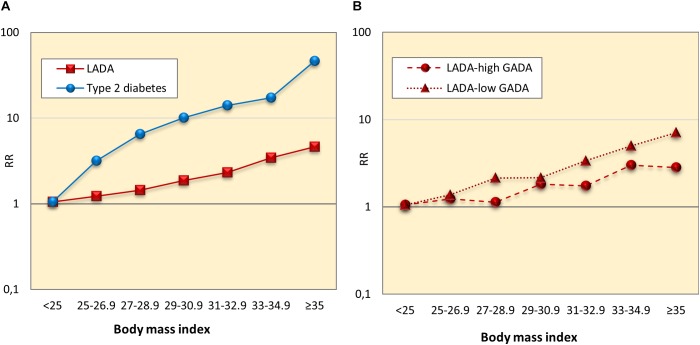
Relative risk of LADA and type 2 diabetes **(A)**, and LADA with high and low GADA levels **(B)** in relation to Body mass index.

Findings of the studies on lifestyle and risk of LADA compared to type 2 diabetes conducted to date are summarized in Figure 1. In support of a role for insulin resistance and a type 2-like etiology, these studies indicate that LADA may share several risk factors with type 2 diabetes; Excess risk of LADA is seen in relation to overweight (Hjort et al., 2018b), adiposity (Hjort et al., 2018b), low birth weight (Hjort et al., 2015), and sweetened beverage intake (Löfvenborg et al., 2016), whereas the risk is reduced in those with moderate alcohol consumption (Rasouli et al., 2013a, 2014) and high physical activity (Carlsson et al., 2007; Hjort et al., 2018a; Figure 1). The associations are generally weaker for LADA than for type 2 diabetes (Figure 1), which is not surprising since insulin resistance can be expected to be less important in disease progression in the presence of autoimmunity and a more pronounced insulin deficiency. This is particularly evident for BMI; as shown in Figure 2, data from the ESTRID study (data set described in Hjort et al., 2018b) indicate that the risk of both LADA and type 2 diabetes increases progressively with BMI but more dramatically for the latter. Furthermore, the excess risk of LADA conferred by high BMI is stronger for less autoimmune LADA (GADA < median), even though it is also seen for more autoimmune LADA (GADA > median) (Figure 2). This is in line with findings of cross-sectional studies, including the NIRAD (Non-Insulin Requiring Autoimmune Diabetes) (Buzzetti et al., 2007) and Action LADA (Hawa et al., 2013) studies showing that LADA patients with low GADA have higher BMI and higher prevalence of the metabolic syndrome than those with high GADA levels.

**FIGURE 3:**
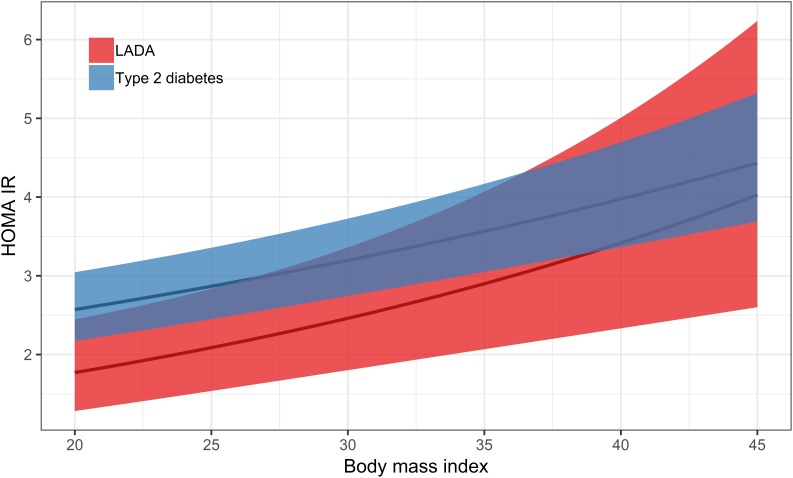
Association between BMI and HOMA-IR in LADA and type 2 diabetes. The curved line represents results of a regression and the shaded surface represents 95% confidence intervals. Data from the ESTRID-study (data set described in Hjort et al., 2018b).

**FIGURE 4:**
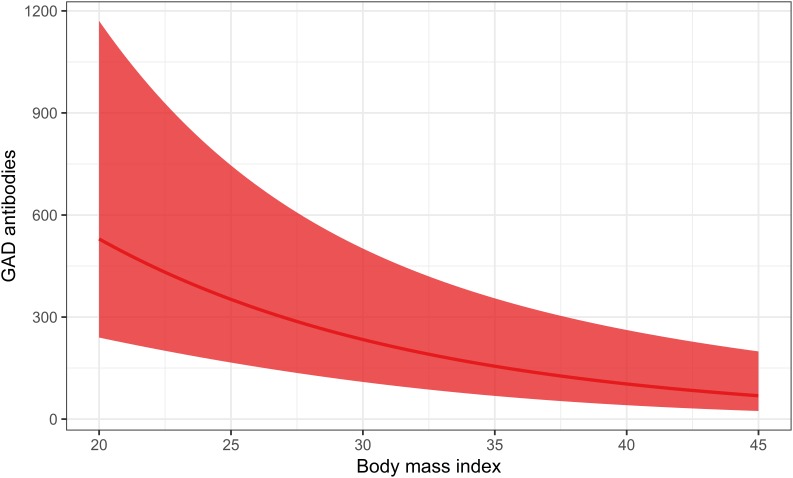
Association between BMI and GADA levels in LADA. The curved line represents results of a regression and the shaded surface represents 95% confidence intervals. Data from the ESTRID-study (data set described in Hjort et al., 2018b).

With regard to underlying mechanisms, results based on the ESTRID Study indicate that BMI (Hjort et al., 2018b) and smoking (Rasouli et al., 2016) are positively associated with insulin resistance calculated by homeostasis model assessment (HOMA-IR) (The Diabetes Trials Unit, 2017) in LADA, with a similar non-significant tendency for sweetened beverage intake (Löfvenborg et al., 2016), whereas there is an inverse association between alcohol consumption (Rasouli et al., 2014), physical activity (Hjort et al., 2018a) and insulin resistance (Table 2). In contrast, there is no indication that the mechanism linking any of these factors to LADA includes a triggering or exacerbating effect on autoimmunity (Table 2). Figure 3, 4 displays the association between BMI and HOMA_IR and GADA in LADA and type 2 diabetes based on the ESTRID-study (data set described in Hjort et al., 2018b). These data indicate that BMI is positively associated with HOMA_IR both in patients with LADA and type 2 diabetes (Figure 3) which fits with previous observations (Chiu et al., 2007). GADA, on the other hand, was inversely associated with BMI among LADA patients (Figure 4).

**Table 2:** Lifestyle factors and associations with risk of LADA, insulin resistance and GADA, results based on HUNT and ESTRID studies.

		Norwegian HUNT Study	Swedish ESTRID Study		
		Association with LADA incidence	Association with LADA incidence	Association with insulin resistance^∗^	Association with GADA levels^∗^
*Increased risk*	Overweight/obesity (Hjort et al., 2018b)	↑	↑	↑	↓
	Low birth weight (Hjort et al., 2015)		↑		
	Smoking (Rasouli et al., 2013b, 2016)	↓	↑	↑	↓
	Coffee (Rasouli et al., 2018)		↑		↑
	Sweetened beverages (Löfvenborg et al., 2016)		↑	↑ ±	
*Reduced risk*	Alcohol (Rasouli et al., 2013a, 2014)	↓	↓	↓	
	Fatty fish (Löfvenborg et al., 2014)		↓		
	Physical activity (Carlsson et al., 2007; Hjort et al., 2018a)	↓	↓	↓	
*No association*	Serious life events (Rasouli et al., 2017b)		→		
	Smokeless tobacco use (Rasouli et al., 2017a)		→		


*↑, increased risk; ↓, reduced risk; →, no association. ^∗^,↑, Positive association or higher mean/median level in exposed than unexposed; ↓, Negative association or lower mean/median in exposed than unexposed. ± HOMA-IR 3.7 vs. 2.5, *P* = 0.0561 in high (>2 servings per day) compared to low consumers*.

##### Environmental factors in LADA associated with autoimmunity and type 1 diabetes

What triggers autoimmunity besides genetic factors is not clear; Psychological stress has been linked to type 1 diabetes in children (Rewers and Ludvigsson, 2016) but no such association was seen with LADA (Rasouli et al., 2017b). In line with some findings regarding type 1 diabetes in children (Stene et al., 2003; Norris et al., 2007; Niinistö et al., 2017), fatty fish is associated with a reduced risk of LADA but unrelated to type 2 diabetes in the ESTRID Study (Löfvenborg et al., 2014). A beneficial effect could hypothetically be attributed to omega 3-fatty acids which are abundant in fatty fish and possessing anti-inflammatory and immunomodulatory properties (Calder, 2013). Moreover, coffee consumption is positively associated with the risk of LADA in carriers of HLA high risk genotypes, as well as with GADA levels (Rasouli et al., 2018), which is in contrast with the reduced risk of type 2 diabetes consistently shown in high consumers of coffee (Carlström and Larsson, 2018). This may be a spurious finding but interestingly, an earlier study on type 1 diabetes with onset in adolescence showed a similar association (Virtanen et al., 1994). A mechanism remains to be established but as outlined in a recent review, several components of coffee may have immunomodulatory effects (Sharif et al., 2017). Since the HUNT Study has limited information on dietary factors, these findings are solely based on data from the ESTRID Study and should clearly be interpreted with caution.

##### Inconsistencies

Results were consistent across the HUNT and ESTRID-studies for BMI, physical activity and alcohol intake (Table 2) but not for smoking which was associated with a significantly increased risk in the Swedish data (Rasouli et al., 2016) and a reduced risk in the Norwegian data (Rasouli et al., 2013b). Previous studies have shown that whereas smoking is associated with an increased risk of type 2 diabetes, primarily attributed to negative effects of nicotine on insulin sensitivity, several studies have linked parental smoking to a reduced risk of type 1 diabetes in the offspring (Dahlquist and Källén, 1992; Magnus et al., 2018), including a recent study on maternal smoking during pregnancy based on data from three different cohorts (Magnus et al., 2018). The authors speculate that a beneficial influence may be due to immune suppressive effects of nicotine (Magnus et al., 2018). Interestingly, in LADA patients, smoking is positively associated with insulin resistance and negatively associated with GADA levels (Rasouli et al., 2013b, 2016). This indicates that smoking may confer both positive and negative effects and population characteristics including degree of underlying autoimmunity may determine whether the net effect is beneficial or detrimental. The effects may also cancel out each other which may explain why we did not find an association between use of Swedish oral moist snuff and LADA (Rasouli et al., 2017a). Interaction with genetic factors may account for some of this heterogeneity; notably, strong interaction between smoking and HLA genotypes has been demonstrated in relation to rheumatoid arthritis (Kallberg et al., 2007) but this remains to be explore in relation to LADA.

## Discussion

**FIGURE 5:**
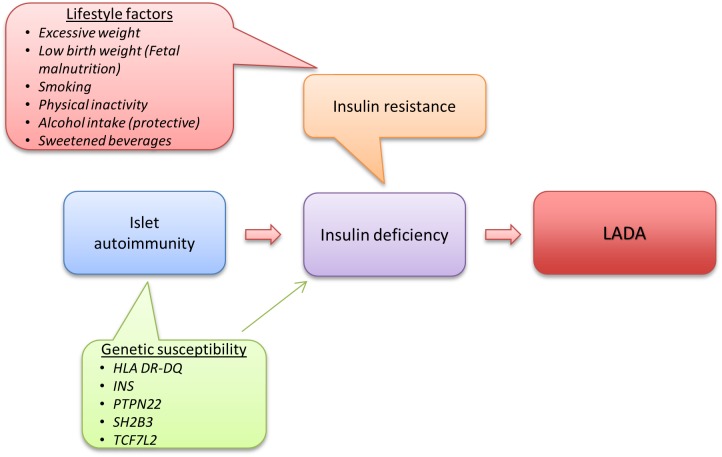
Etiology of LADA, model based on current knowledge.

Despite the autoimmune nature of LADA and clear genetic overlap with type 1 diabetes, studies on lifestyle and LADA risk indicate that factors such as overweight and physical inactivity that are associated with insulin resistance and type 2 diabetes may also promote LADA. This indicates that insulin resistance may play a key role in the pathogeneses of LADA together with autoimmune destruction of the insulin producing beta-cells. Based on current knowledge, one can create a model over LADA development where the first step in disease development is genetically triggered autoimmunity that slowly destroys the beta-cells and reduces insulin release. At a second stage, exposure to unhealthy lifestyle factors leads to insulin resistance and increased demand on the beta-cells for a compensatory rise in insulin production. Eventually the beta-cells will fail to meet the increasing insulin need, resulting in hyperglycemia and LADA becomes manifest (Figure 5).

**FIGURE 6:**
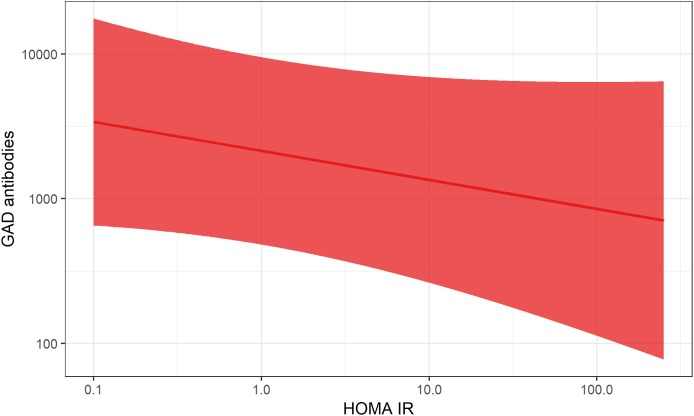
Association between HOMA-IR and GADA levels in LADA. The curved line represents results of a regression and the shaded surface represents 95% confidence intervals. Data from the ESTRID-study (data set described in Hjort et al., 2018b).

This fits with the accelerator hypothesis (Wilkin, 2001), which proposes that insulin resistance may be involved in the promotion of all forms of diabetes by stressing the beta-cells, possibly accelerating the autoimmune process as well as beta-cell apoptosis. In line with this reasoning, excessive weight has also been linked to the risk of type 1 diabetes in children (Verbeeten et al., 2011). The relative importance of insulin resistance for disease progression will most likely depend on the degree of underlying autoimmunity; if autoimmunity is severe enough, the influence of factors promoting insulin resistance is most likely minor. Consistent with this hypothesis, data from the ESTRID Study (Hjort et al., 2018b) show a negative association between GADA and HOMA-IR in LADA patients at time of diagnosis (Figure 6). This also fits with reasoning by Naik et al. who proposed that insulin resistance will determine at what point in the autoimmune process LADA will become manifest (Naik et al., 2009).

### Opportunities for Prevention

Intervention studies show that it is possible to prevent type 2 diabetes by lifestyle modification (Tuomilehto et al., 2001; Knowler et al., 2002). In the Finnish Diabetes Prevention Study and the United States Diabetes Prevention Program, a 58% risk reduction among individuals with pre-diabetes was achieved by diet modification (e.g., increase in fiber intake and reduction of fat intake) and increased physical activity, with weight loss as the key factor behind the protective effect. A partly similar etiology of LADA suggests that it may also be possible to prevent or postpone LADA through the same lifestyle modifications. The fact that GADA has been detected in LADA patients several years prior to diagnosis suggests that LADA, similar to type 2 diabetes, has a long pre-diabetic phase (Lundgren et al., 2010; Sørgjerd et al., 2012) during which it may be possible to intervene. The preventive potential can be expected to be smaller for LADA than for type 2 diabetes where insulin resistance is the main driver of disease development. In line with this, the population attributable fraction of cases attributed to overweight/obesity in the ESTRID Study was estimated at 31% for LADA and 82% for type 2 diabetes (Hjort et al., 2018b). In order to investigate whether lifestyle intervention may reduce LADA risk, intervention studies are needed and such studies could target people with high risk, e.g., those with high genetic risk or antibody positivity similar to what has been done in type 1 diabetes (Skyler, 2013). Unfortunately, it has proved to be very difficult to prevent type 1 diabetes in children (Skyler, 2013). However, the trials conducted to date have primarily targeted factors with hypothesized effect on development and progression of autoimmunity and not insulin resistance which could prove to be fruitful in the prevention of LADA. Consistent with findings in children, adult carriers of autoantibodies are at increased risk of developing diabetes (Lundgren et al., 2010), and screening of non-diabetic individuals indicate that 0.7–4.7% of the general population display such autoantibody positivity (Ruige et al., 1997; Lundgren et al., 2010; Rolandsson et al., 2015; Sørgjerd et al., 2015). Such individuals may be suitable targets for intervention studies, under the assumption that whether a person acquires insulin resistance determines if and when the autoimmune reaction will result in hyperglycemia. Furthermore, preliminary findings based on the ESTRID and HUNT studies (Hjort et al., 2017b) indicate that the combination of HLA genotypes, in particular DR4/4, and overweight dramatically increases the risk of LADA. Carriers of these genotypes may also be considered for intervention studies focusing on weight reduction.

### Much Remains to Be Explored

At this point it has to be stressed that current knowledge of the role of lifestyle factors in the etiology of LADA is still very limited. There are few studies and they are all based on data from two Scandinavian studies. The HUNT and ESTRID studies have limitations; HUNT has the advantage of being a prospective cohort study, at the same time the number of patients is small. ESTRID on the other hand, is based on more than twice as many cases but uses a case-control design which is more efficient for rare conditions, but implies that lifestyle information is collected retrospectively and this may introduce recall bias. Strengths of the studies include the detailed information on lifestyle and genetic information, careful characterization of cases and population-based design. Moreover, the consistency of findings across the two cohorts provides support for the validity of the data at hand. Interestingly, data from the Swedish ESTRID-study suggests a protective effect of fatty fish (Löfvenborg et al., 2014) and a harmful effect of coffee consumption (Rasouli et al., 2018) on the risk of LADA, that is not seen for type 2 diabetes. Moreover, findings regarding smoking seemed to go in opposite directions in the Swedish (Rasouli et al., 2016) and Norwegian data (Rasouli et al., 2013b). Replications of these findings in other populations are clearly warranted and there are many potential risk factors that remain to be investigated. This is especially true for dietary factors. Moreover, gene^∗^environment interaction in LADA is an important but currently unexplored field. Since lifestyle factors are likely to act on genetic susceptibility in the promotion of LADA, this is important to address if we want to understand how LADA develops.

### What About Prognosis?

So what does the specific pathogenesis of LADA entail in terms of prognosis and disease management? Data from a small number of studies indicate that LADA patients have worse glycemic control than patients with type 2 diabetes (Olsson et al., 2013; Hawa et al., 2014) which may be due to the limited endogenous insulin production. Lack of established treatment guidelines may also play a role; as outlined in a recent review there is a shortage of randomized clinical trials in LADA and the optimal treatment regimen is still unknown (Hals, 2018). Moreover, in the absence of autoantibody testing, LADA patients are likely to be diagnosed as having type 2 diabetes and treated according to such recommendations, which may not be optimal. The combination of poor glycemic control and insulin resistance, together with other features of the metabolic syndrome may put individuals with LADA at high risk of complications. As summarize in a recent review (Buzzetti et al., 2017), data from the few studies conducted to date indicate that the risk of both micro- and macro vascular complications is at least as high in LADA as in type 2 diabetes patients, in spite of their generally healthier metabolic profile. The long term consequences of LADA and the role of different prognostic factors such as treatment and lifestyle are important to elucidate in order to improve secondary prevention. As pointed out by Buzzetti et al. (2017), future studies need to take into account the heterogeneous nature of LADA; the degree of underlying autoimmunity and subsequent beta-cells loss will most likely affect response to treatment as well as prognosis.

## Conclusion

Taken together, studies conducted to date indicate that LADA, similar to type 2 diabetes, results from an imbalance between insulin sensitivity and insulin secretion. However, the relative contribution of insulin deficiency to disease progression is greater in LADA and of different origin, namely due to the same autoimmune pathogenesis underlying type 1 diabetes. The findings of an association between unhealthy lifestyle factors and LADA opens up the possibility that LADA to some extent may be prevented through the same lifestyle modifications as type 2 diabetes, including healthy diet, increased physical activity and subsequent weight loss. Intervention studies to test this hypothesis are needed and such studies may proposedly target GADA positive individuals without manifest diabetes or individuals with high genetic risk.

## Data Availability

The datasets for this manuscript are not publicly available because the datasets analyzed during the current study are available from the corresponding author on reasonable request (ESTRID) and with permission of the HUNT Study by applying to the HUNT Study data access committee. Requests to access the datasets should be directed to sofia.carlsson@ki.se.

## Author Contributions

SC conceived and wrote the manuscript and agreed to be accountable for its content.

## Conflict of Interest Statement

The author declares that the research was conducted in the absence of any commercial or financial relationships that could be construed as a potential conflict of interest.
